# Fermentative Production of *N*-Methylglutamate From Glycerol by Recombinant *Pseudomonas putida*

**DOI:** 10.3389/fbioe.2018.00159

**Published:** 2018-11-09

**Authors:** Melanie Mindt, Tatjana Walter, Joe Max Risse, Volker F. Wendisch

**Affiliations:** ^1^Genetics of Prokaryotes, Faculty of Biology and CeBiTec, Bielefeld University, Bielefeld, Germany; ^2^Fermentation Technology, Technical Faculty and CeBiTec, Bielefeld University, Bielefeld, Germany

**Keywords:** *Pseudomonas putida*, *N*-methylglutamate, *Methylobacterium extorquens*, γ-glutamylmethylamide synthetase, GS/GOGAT, *N*-methylglutamate synthase, GlpR, fed-batch fermentation

## Abstract

*N*-methylated amino acids are present in diverse biological molecules in bacteria, archaea and eukaryotes. There is an increasing interest in this molecular class of alkylated amino acids by the pharmaceutical and chemical industries. *N*-alkylated amino acids have desired functions such as higher proteolytic stability, enhanced membrane permeability and longer peptide half-lives, which are important for the peptide-based drugs, the so-called peptidomimetics. Chemical synthesis of *N*-methylated amino acids often is limited by incomplete stereoselectivity, over-alkylation or the use of hazardous chemicals. Here, we describe metabolic engineering of *Pseudomonas putida* KT2440 for the fermentative production of *N*-methylglutamate from simple carbon sources and monomethylamine. *P. putida* KT2440, which is generally recognized as safe and grows with glucose and the alternative feedstock glycerol as sole carbon and energy source, was engineered for the production of *N*-methylglutamate using heterologous enzymes from *Methylobacterium extorquens*. About 3.9 g L^−1^ *N*-methylglutamate accumulated within 48 h in shake flask cultures with minimal medium containing monomethylamine and glycerol. A fed-batch cultivation process yielded a *N-*methylglutamate titer of 17.9 g L^−1^.

## Introduction

Alkylated amino acids became the focus of attention by the pharmaceutical and chemical industry over the last years. *N*-alkylated amino acids are used in the design of peptide-based drugs named peptidomimetics in order to enhance their half-lives and stability against proteolysis or their membrane permeability. Peptidomimetics are imitating structures of natural bioactive peptides and have improved pharmaceutical properties (Gentilucci et al., 2010; Di Gioia et al., 2016). These advantages are also present in naturally occurring alkylated amino acids and peptides such as the medically relevant compounds destruxin B or cyclosporine A. Cyclosporine A is one of the most important and medically relevant examples of an *N*-methylated peptide. It shows potent immunosuppressant properties, low toxicity and metabolic stability as a medical drug for organ transplantation (Di Gioia et al., 2016). Destruxin B, isolated from entomopathogenic fungus *Metarhizium anisopliae*, belongs to the cyclodepsipeptides with insecticidal and anticancer activities and was shown to induce apoptosis in human non-small cell lung cancer cells (Wu et al., 2013). Besides the natural *N*-methylated molecules, *N*-methylation was applied to natural bio-active peptides to generate synthetic peptidomimetics. The antitumor agent sansalvamide, which is naturally occurring in the marine fungus of the genus *Fusarium*, was chemically *N*-methylated to generate the *N*-methylsansalvamide, which shows higher potency and selectivity of antitumor activity in comparison to the natural product (Liu et al., 2005). Moreover, *N*-methylation of tubulysin enhanced its antimitotic activity (Patterson et al., 2008).

Nowadays, *N*-methylated amino acids are mainly chemically synthesized. There are three main synthetic strategies for the *N*-methylation of amino acids: (I) the reductive amination according to White and Konopelski (2005) (II) the reductive ring opening of 5-oxazolidinones according to Freidinger et al. (1983) and (III) the use of methylating reagents (Di Gioia et al., 2016). Chemical methods may use hazardous chemicals, give only incomplete stereoselectivity and low yields, while side reactions like dimethylation of the amino group may occur. Bacterial fermentation and heterologous overexpression is a new and efficient production strategy for this interesting class of molecules.

Biosynthesis of *N*-methylated amino acids occurs in all three domains of life to extend the chemical repertoire of the 20 standard amino acids as building blocks to new or altered properties. Bacterial *N*-methyl amino acids for example occur in posttranslationally modified extracellular secretion proteins (Strom et al., 1993), in ribosomal proteins or serve as intermediates of metabolic pathways (Stock et al., 1987; Nesterchuk et al., 2011; Gruffaz et al., 2014).

*N*-methylglutamate (NMeGlu), for example, is an intermediate of the monomethylamine (MMA) catabolism in several Gram-negative bacteria (Gruffaz et al., 2014). MMA is synthesized during the breakdown of proteins and amine osmolytes by anaerobic bacteria that decarboxylate nitrogenous organic compounds. Moreover, it is released during biomass combustion or in industrial processes such as fish processing (Barrett and Kwan, 1985; Bhadbhade et al., 2002; Chen et al., 2010). MMA is ubiquitous in the environment and can function as carbon, energy and/or nitrogen source for diverse bacteria, where its oxidation is an important component of the C and N cycle in the environment (Nayak and Marx, 2014a). Methylotrophs grow on reduced single-carbon compounds like MMA as the sole source of carbon and energy. Gram-positive bacteria and methylotrophic yeasts oxidize MMA in a one-step reaction to formaldehyde by the Cu-containing methylamine oxidase (MAO) (van Iersel et al., 1986; Cai and Klinman, 1994). In Gram-negative bacteria, oxidation of MMA to formaldehyde is catalyzed by the MMA dehydrogenase (MADH), encoded by the *mau* cluster (Chistoserdov et al., 1994). Alternatively, a multistep pathway, the so-called *N*-methylglutamate pathway, operates in some methylotrophic bacteria such as *Methylobacterium extorquens, Methylocella silvestris*, and *Methyloversatilis universalis* (Chen et al., 2010; Latypova et al., 2010; Gruffaz et al., 2014; Nayak and Marx, 2014b).

The *N*-methylglutamate pathway, which based on recent metagenomic data from natural ecosystems is abundant in nature in addition to MMA oxidizing enzymes and was proposed in 1966 by Shaw et al. to explain methylamine-depending growth of several microbial species (Shaw et al., 1966). This was corroborated by genetic analysis of the key enzymes from *Methyloversatilis universalis* FAM5 in 2010 (Latypova et al., 2010). Subsequently, *N*-methylglutamate pathway genes were detected in additional microbes including *Methylocella silvestris* BL2 and *Methylobacterium extorquens* (Chen et al., 2010; Gruffaz et al., 2014; Nayak and Marx, 2014b). In *M. extorquens* DM4, a strain lacking methylamine dehydrogenase, genes encoding enzymes of the *N*-methylglutamate pathway were identified by comparative genomics (Gruffaz et al., 2014). Its genome contains the operon *mgsABC-gmaS* encoding the *N*-methylglutamate synthase (METDI2324, METDI2325, METDI2326; *mgsABC*) and γ-glutamylmethylamide synthetase (METDI2327; *gmaS*) while the adjacent, but divergently oriented *mgdABCD* operon encodes *N*-methylglutamate dehydrogenase (METDI2322, METDI2321, METDI2320, METDI2319). GMAS catalyzes the ATP-dependent methylamidation of l-glutamate. The resulting γ-glutamylmethylamid (GMA) is further converted to NMeGlu by the *N*-methylglutamate synthase (NMGS). In the final step, *N*-methylglutamate dehydrogenase oxidizes NMeGlu to l-glutamate and formaldehyde which is assimilated as C source in the serine cycle (Gruffaz et al., 2014). Null mutations in genes encoding enzymes of the *N*-methylglutamate pathway in *M. extorquens* DM4 showed that GMAS is essential for growth on MMA as a carbon source but not as a nitrogen source. Currently, the redox cofactors of NMGS are unknown and the pathway has not been characterized to biochemical detail. Based on the current genetic understanding of the *N*-methylglutamate pathway, we chose *gmaS* and *mgsABC* to engineer a pathway for fermentative NMeGlu production.

**Figure 1:**
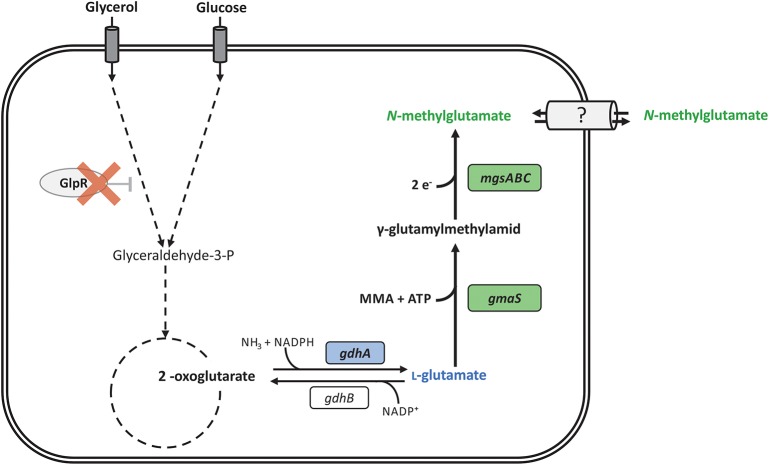
Schematic overview of the reaction catalyzed by γ-glutamylmethylamide synthetase (GMAS) and *N*-methylglutamate synthase (NMGS) and their incorporation in the metabolism of *P. putida* KT2440. *N*-methylglutamate formation (thick arrows) takes place by heterologous expression of *gmaS* and *mgsABC* from *M. extorquens* DM4 (green boxes) starting from l-glutamate. Deletion of the transcriptional repressor gene of the glycerol metabolism (*glpR*) is indicated by red cross. The second copy of endogenous glutamate dehydrogenase (*gdhA*) under control of Ptac was integrated in *glpR* locus (blue boxes). Reactions are indicated by an arrow, multiple reactions by dashed arrows.

As production host we chose the GRAS organism *P. putida* KT2440 since its genome is fully sequenced, genetic tools are available and it is relevant for several fermentation processes due to its high tolerance to solvents and oxidative stress and its capability to degrade aromatic compounds (Nikel et al., 2014b; Loeschcke and Thies, 2015). Interestingly, *P. putida* KT2440 grows well with glycerol, an attractive raw material for fermentation and available as dominant by-product of the biodiesel production (Solaiman et al., 2006; Escapa et al., 2013). For instance, production of PHA from crude glycerol as carbon source was demonstrated (Poblete-Castro et al., 2014). Here, we describe metabolic engineering of *Pseudomonas putida* KT2440 for the fermentative production of *N*-methylglutamate from glycerol and MMA (Figure 1).

## Materials and methods

### Bacterial strains and growth conditions

Microorganisms and plasmids used in this study are listed in Table 1. *E. coli* DH5α was used for gene cloning and *P. putida* KT2440 was used as platform strain for metabolic engineering. Pre-cultivation of *E. coli* was performed in lysogeny broth (LB) and of *P. putida* in LB or in M12 minimal medium with 20 g L^−1^ glycerol inoculated from a fresh LB agar plate. For growth and production experiments of *P. putida* strains, the pre-culture cells were washed once in TN buffer (pH 6.3, 50 mM Tris, 50 mM NaCl) without carbon source and inoculated to an initial optical density (OD_600_) of 0.5. Standard cultivations were performed in M12 minimal medium in the presence of 20 g L^−1^ glycerol or glucose as carbon source in 500 mL baffled flaks on a rotary shaker (120 rpm) at 30°C. *E. coli* was routinely cultivated in LB in 500 mL baffled flasks at 37°C on a rotary shaker (180 rpm). Gene expression from the pEV1 based plasmids was induced by addition of 0.5 g L^−1^ l-rhamnose. When necessary, the growth medium was supplemented with tetracycline (10 μg mL^−1^ for *E. coli* and 50 μg mL^−1^ for *P. putida*).

**Table 1:** Strains and vectors used in this work.

**Strains and vectors**	**Description**	**Source**
**STRAINS**		
*P. putida* KT2440	*P. putida* mt-2 hsdRl hsdM	Bagdasarian et al., 1981; Franklin et al., 1981
*P. putida* KT2440 *Δupp*	*P. putida* mt-2 hsdRl hsdM *Δupp*	Graf and Altenbuchner, 2011
*P. putida* KT2440 *Δupp ΔglpR*::Ptac-*gdhA*	*P. putida* mt-2 hsdRl hsdM *Δupp ΔglpR*::Ptac-*gdhA*	This work
*E. coli* DH5α	F-*thi-*1 *endA1 hsdr17*(r-, m-) *supE44* Δ*lacU169* (Φ80*lacZ*ΔM15) *recA1 gyrA96*	Hanahan, 1983
*M. extorquens* DM4	Wild type DM4	Gälli and Leisinger, 1985; Vuilleumier et al., 2009
NMG0	*P. putida* KT2440 carrying pEV1	This work
NMG1	*P. putida* KT2440 carrying pEV1-*mgsABC*	This work
NMG2	*P. putida* KT2440 carrying pEV1-*mgsABC-gmaS*	This work
NMG3	*P. putida* KT2440 *Δupp ΔglpR*::Ptac-*gdhA* carrying pEV1-*mgsABC-gmaS*	This work
**VECTORS**		
pEV1	Expression shuttle vector pACYC184 derivative containing TetR, *pVS1_ori, p15A_ori*,*rha*P_BAD_ expression system	Provided by Evonik Creavis GmbH
pEV1-*mgsABC-gmaS*	pEV1 derivative for l-rhamnose inducible expression of *mgsABC-gmaS* from *M. extorquens* DM4 containing an artificial RBS 5′-CTAGGAGGATTCGTC-3′	This work
pEV1-*mgsABC*	pEV1 derivative for l-rhamnose inducible expression of *mgsABC* from *M. extorquens* DM4 containing artificial RBS 5′-CTAGGAGGATTCGTC-3′	This work
pKOPp	pJOE6261.2 derivative for deletions in *P. putida* KT2440	Graf and Altenbuchner, 2011
pKOPp-*glpR*::Ptac-*gdhA*	pJOE6261.2 derivative for deletion of *glpR* in *P. putida* KT2440 and integration of *gdhA* containing an artificial RBS 5′-CTAGGAGGATTCGTC-3′ under the control of the constitutive promotor Ptac	This work

### Molecular biological techniques

Standard molecular techniques were performed as described elsewhere (Green and Sambrook, 2012). Competent *E. coli* DH5α (Hanahan, 1983) cells were prepared according to the RbCl method and the transformation was performed by heat-chock at 42°C for 45 s (Green and Sambrook, 2012). *P. putida* cells were washed with 10% (v/v) glycerol and transformed via electroporation (2.5 kV, 200 W, and 25 F) according to published methods (Iwasaki et al., 1994). Extraction of genomic DNA of *Methylobacterium extorquens* DM4 (DSM6343) by precipitation was based on Eikmanns et al. (1994). Preparation of plasmid DNA from *E. coli* and *P. putida* was performed using GeneJet Plasmid Miniprep Kit from Thermo Scientific with the protocol according to the manufacturer. PCR amplification was performed with Phusion High-Fidelity DNA polymerase and ALLin™ HiFi DNA Polymerase according to the manufacturer (New England Biolabs, UK or highQu GmbH, GER). PCR clean-up was done according to the protocol of GeneJET PCR Purification Kit and GeneJET Gel Extraction Kit of Thermo Fisher Scientific (USA). Restriction enzymes were purchased from Thermo Fisher Scientific (USA). The isothermal assembly method for assembly of multiple fragments were used as described previously (Gibson et al., 2009). All cloned DNA fragments were confirmed by DNA sequence analysis (Sequencing Core Facility, Bielefeld University).

The operons *mgsABC-gmaS* and *mgsABC* were amplified from *M. extorquens* DM4, using the primers P1, P2, and P3 (see Supplementary Table S1). The purified PCR fragments were cloned into *Kpn*I/ *Sda*I digested plasmid pEV1, which led to the plasmids pEV1-*mgsABC* and pEV1-*mgsABC-gmaS* (Table 1).

### Chromosomal gene replacement

Gene replacements in *P. putida* were performed by homologous recombination for marker less gene deletions as described before (Graf and Altenbuchner, 2011) by using the suicide vector pKOPp. The *P. putida* gene sequences for the suicide vector pKOPp-Δ*glpR::*P*tac-gdhA*, were amplified using the primers P4 and P5 (500 pb upstream of *glpR*), P6 and P7 (cds of *gdhA* gene) and P8 and P9 (500 bp downstream of *glpR*) (see Supplementary Table S1). Screening of the mutants was performed on LB agar supplemented with 0.04 mg mL^−1^ 5-fluorouracil. The replacement of the *glpR* gene was verified by using the primer P10 and P11 (see Supplementary Table S1).

### Crude extract preparation and protein analysis

Cultures for protein gel or enzyme assays were inoculated as described above in minimal medium with either glucose or glycerol as carbon source. Cells were harvested after 24 h and stored at −20°C for later analysis. From this step, cells and crude extracts were kept at 4°C or on ice. The cells were resuspended in 1 mL 200 mm KP_i_ and sonicated (UP 200 S, Dr. Hielscher GmbH, Teltow, GER) for 3 min at an amplitude of 60% and a duty cycle of 0.5. After centrifugation (20200 × g, 45 min, 4°C) protein concentration of the cell-free extract was obtained by Bradford-method (Bradford, 1976) using bovine serum albumin as reference. Sodium dodecyl sulfate polyacrylamide electrophoresis (SDS-PAGE) was performed as described elsewhere (Green and Sambrook, 2012) and 10 μg of protein sample was used.

Determination of glutamate dehydrogenase activity was performed according to Meers et al. (1970) and consumption of NADPH (ϵ = 6200 L mol^−1^ cm^−1^) was followed at 340 nm at 30°C for 4 min. The assay was performed at least in triplicates.

### Culture conditions

Growth of *P. putida* KT2440 in presence of MMA was performed in the Biolector microfermentation system (m2p-labs, Aachen, GER) at 30°C and a shaking frequency of 1200 rpm. Cells were cultivated in 48-well flower plates in 1 mL minimal medium and growth was followed by backscattered light at 620 nm and a signal gain factor of 20.

Production experiments were performed in 500 mL baffled shaking flasks containing 50 mL M12 minimal medium with 20 g L^−1^ of carbon source, 100 mM MMA with 0.5 g L^−1^ l-rhamnose and addition of antibiotics. The cultures were cultivated for 48 h at 30°C with 120 rpm. For production on glucose or glycerol, the main cultures were inoculated to OD_600_ 0.5 using washed cells of LB pre-cultures. In addition, the minimal medium was supplemented with 0.5 g L^−1^ l-rhamnose and 50 μg mL^−1^ tetracycline. Production was determined regularly by HPLC analysis.

### Fed-batch cultivation of *P. putida* KT2440 strains

Production of NMeGlu by strain NMG3 was performed in a fed-batch cultivation process with an initial volume of 2 L in a bioreactor (3.7 L KLF, Bioengineering AG, SUI) at 30°C and 0.2 bar overpressure. Aeration rate was set to 2 NL min^−1^ and to sustain a relative dissolved oxygen saturation (rDOS) of 30%, the stirrer speed was controlled. By controlled addition of phosphoric acid [10% (w/w)] and ammonia [25% (w/w)] a pH 7.0 was maintained. To avoid foaming Pluronic PE 8100 was added when necessary. Addition of the feed medium (600 g L^−1^ glycerol, 100 g L^−1^ yeast extract and 1 g L^−1^ MgSo${{}_{4}}^{*}$ H_2_O) was depending on the relative dissolved oxygen saturation when the rDOS signal exceeded 60% and stopped when rDOS felt below 60%. Beginning with cultivation start, 0.5 g L^−1^ l-rhamnose was added once and 100 mM MMA (according to the initial volume) was added at the beginning and at 24 and 48 h. Samples were taken automatically in appropriate intervals and cooled to 4°C until analysis. Biomass formation, feed volume and all concentrations were related to the initial volume. For the batch phase HSG medium was used: 13.5 g L^−1^ soy peptone, 7 g L^−1^ yeast extract, 20 g L^−1^ glycerol, 2.5 g L^−1^ NaCl, 2.3 g L^−1^ K_2_HPO_4_, 1.5 g L^−1^ KH_2_PO_4_, and 0.14 g L^−1^ MgSo${{}_{4}}^{*}$H_2_O. The bioreactor was inoculated by addition of 100 mL of an overnight culture of NMG3 in HSG medium containing 50 μg mL^−1^ tetracycline.

### Analytical quantification of amino acids and carbohydrates

Extracellular amino acids and organic acids were quantified by the analytical high-performance liquid chromatography (HPLC, 1200 series, Agilent Technologies Deutschland GmbH, GER). For quantification, the supernatant of production experiments was used. The derivatization with 9-fluorenylmethyl chloroformate (FMOC) according to published methods (Schneider et al., 2012) with modifications (Jensen and Wendisch, 2013) was performed for detection of primary and secondary amines. The separation was performed with a pre-column (LiChrospher 100 RP8 EC-5μ (40 × 4.6 mm), CS-Chromatographie Service GmbH, GER) and a main column (LiChrospher 100 RP8 EC-5μ (125 × 4.6 mm), CS-Chromatographie Service GmbH, GER). The detection was performed with a fluorescence detector (FLD G1321A, 1200 series, Agilent Technologies, USA) with excitation and emission wavelength of 263 and 310 nm respectively.

Derivatization and quantification were carried out with the following modifications for the quantification of FMOC derivatized samples: l-isoleucine was used for derivatization instead of l-glycine and l-proline was used as internal standard. The used mobile phases were A: 50 mM sodium acetate (pH 4.2) and B: acetonitrile with the gradient: 0 min 38% B, 5 min 38% B, 12 min 57% B, 14 min 76% B, 15 min 76% B, and 18 min 38% B.

The organic acid 2-oxoglutarate was separated with a column for organic acids (Aminex 300 × 8 mm, 10 μm particle size, 25 Å pore diameter, CS-Chromatographie Service GmbH, GER) under isocratic conditions for 17 min at 60°C with 5 mM sulfuric acid and a flow rate of 0.8 mL min^−1^. The Diode Array Detector (DAD, 1200 series, Agilent Technologies) at 210 nm was used for molecule detection.

Glycerol was determined by ion exchange HPLC as described elsewhere (Müller et al., 2013). The analytical procedure takes place with an ion exchange column (Nucleogel Sugar 810 H, 300 × 7.8 mm, 10 μm particle size, 100 Å pore diameter, Macherey-Nagel, GER) under isocratic conditions for 25 min at 72°C with 2.5 mM sulfuric acid and a flow rate of 0.8 mL min^−1^. The refractive index detector (RID, Irica ERC-7515 A, Erma CR. Inc.) at 210 nm was used for molecule detection.

## Results

### Establishing fermentative production of NMeGlu in *P. putida*

**Figure 2:**
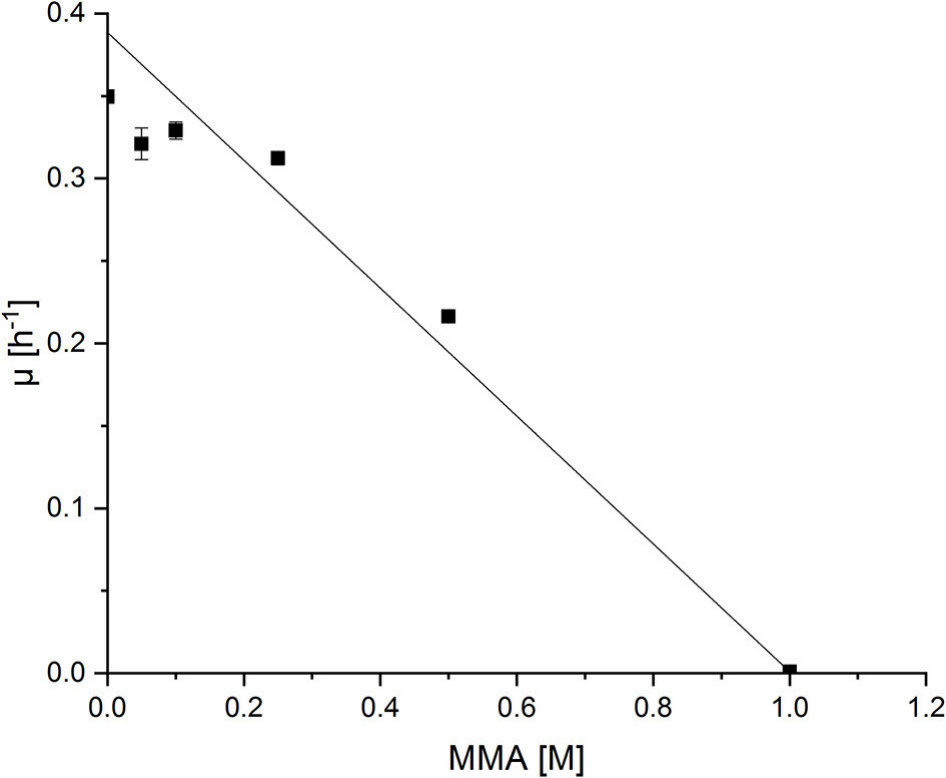
Growth rates of *P. putida* KT2440 grown in the presence of varying concentrations of MMA. *P. puida* KT2440 wild type strain was grown in minimal medium supplemented with increasing MMA concentrations (0.05 m – 1.0 m) and growth rates were determined. Half maximal effect on growth rate was determined by linear fit ting (OriginLab, Northampton, MA).

Production of NMeGlu, an intermediate in the MMA oxidation pathway of *M. extorquens* DM4, by *P. putida* has not yet been described. To test if *P. putida* KT2440 tolerates high MMA concentrations, the substrate of the pathway leading to NMeGlu, the effect of increasing MMA concentrations (0.05 m up to 1.0 m) on the growth behavior of *P. putida* was analyzed (Figure 2). The growth rate was about half maximal at about 0.50 m MMA in minimal medium supplemented with glucose as carbon source. Additionally, the final optical density at 600 nm (OD_600_) diminished at higher MMA concentrations. Thus, *P. putida* KT2440 appeared as host suitable for production of NMeGlu.

Inspection of the genome sequence of *P. putida* KT2440 revealed that no homologs of enzymes for biosynthesis of NMeGlu (GMAS and NMGS) are encoded. The *P. putida* KT2440 enzyme with the highest amino acid sequence similarity to GMAS was a glutamine synthetase. The enzymes with the highest similarity to NMGS on the protein level was a FMN-binding glutamate synthase family protein and a glutamine amidotransferase (see Supplementary Table S2). Thus, *mgsABC* (coding for NMGS) alone or in combination with *gmaS* (coding for GMA synthetase) from *M. extorquens* DM4 were cloned into the shuttle vector pEV1. Considering the proposal that NMGS synthesizes NMeGlu directly from MMA and glutamate, thus, bypassing GMA formation (Gruffaz et al., 2014), the strains *P. putida*(pEV1-*mgsABC*) and *P. putida*(pEV1-*mgsABC-gmaS*) were constructed and designated as NMG1 and NMG2, respectively. Protein gel analysis of strain NMG2 revealed successful recombinant gene expression (see Supplementary Figure S1). These strains were tested for NMeGlu production in minimal medium with 20 g L^−1^ glucose and 100 mM MMA. Heterologous expression of *mgsABC* alone resulted in NMeGlu production. However, NMeGlu production was 2.5 times higher upon heterologous expression of the operon *mgsABC-gmaS* and a titer of 0.5 ± 0.1 g L^−1^ resulted (Table 2). Since only traces of the intermediate GMA (< 0.1 g L^−1^) were detected, biosynthesis of NMeGlu appeared to operate efficiently in the two-step cascade.

**Table 2:** Production of *P. putida* KT2440 strains.

**Strain**	**C source**	**N source**	**2-oxoglutarate [g L^−1^]**	**l-Glu [g L^−1^]**	**GMA [g L^−1^]**	**NMeGlu [g L^−1^]**
NMG1	Glucose	2.2	< 0.5	< 0.1	< 0.1	0.2 ± 0.1
NMG2	Glucose	2.2	< 0.5	< 0.1	< 0.1	0.5 ± 0.1
NMG2	Glycerol	2.2	< 0.5	< 0.1	< 0.1	1.6 ± 0.1
NMG2	Glycerol	3.3	< 0.5	< 0.1	< 0.1	2.9 ± 0.1
NMG3	Glycerol	3.3	< 0.5	< 0.1	< 0.1	3.9 ± 0.1

*Cells were grown in minimal medium supplemented with either 20 g L^−1^ glucose or glycerol and 100 mM MMA and 0.5 g L^−1^l-rhamnose for gene expression. Ammonium sulfate was used as N source at the indicated concentrations in g L^−1^. After 48 h the culture supernatants were harvested and analyzed by HPLC. Production yields are given as means with deviations of three replicates. The detection limit was 0.1 g L^−1^ for the amines l-glutamate and GMA and 0.5 g L^−1^ for 2-oxoglutarate*.

### Metabolic engineering for glycerol-based NMeGlu production

Since glycerol is an alternative feedstock readily used by *P. putida*, glycerol-based production of NMeGlu by *P. putida* NMG2 was tested in M12 minimal medium supplemented with 20 g L^−1^ glycerol and 100 mM MMA. As compared to glucose, the NMeGlu tripled to 1.6 ± 0.1 g L^−1^. Traces of the precursor l-glutamate (< 0.1 g L^−1^), but no 2-oxoglutarate was detected in culture supernatants. The product NMeGlu contains two nitrogen atoms: one introduced via the added MMA and one in the l-glutamate synthesized by glutamate dehydrogenase during growth in minimal medium M12. However, this medium has been developed for growth, but not for overproduction of nitrogenous products. Therefore, it was tested whether increasing the nitrogen concentration of the minimal medium M12 from 2.2 g L^−1^ to 3.3 g L^−1^ ammonium sulfate affected NMeGlu production. As anticipated, the NMeGlu titer increased to 2.9 ± 0.1 g L^−1^ (Table 2).

In order to improve provision of l-glutamate as immediate precursor of NMeGlu production, we targeted amination of 2-oxoglutarate to l-glutamate catalyzed by glutamate dehydrogenase. In a similar manner as described for l-glutamate overproducing *Corynebacterium glutamicum* (Börmann et al., 1992; Kawahara et al., 1997; Asakura et al., 2007; Jorge et al., 2017), we overexpressed the endogenous glutamate dehydrogenase gene *gdhA*. To generate a genetically stable strain, we sought chromosomal integration of *gdhA*. Deliberately, we chose the locus of *glpR* since GlpR functions as transcriptional repressor of glycerol catabolism genes and its deletion shortens the lag phase of glycerol cultures (Nikel et al., 2015). The strong Ptac promoter was used to drive expression of a second copy of the endogenous *gdhA* and two-step homologous recombination in *P. putida* Δ*upp* was used to replace the open reading frame of *glpR* with Ptac*-gdhA*. The resulting strain was named *P. putida* Δ*upp* Δ*glpR*::Ptac*-gdhA* (Figure 1). As expected from previous work using the artificial promoter Ptac in *P. putida* (Swift et al., 2001; Walker and Keasling, 2002; Dammeyer et al., 2011), *P. putida* Δ*upp* Δ*glpR*::Ptac*-gdhA* showed a slightly, but significantly increased glutamate dehydrogenase activity (21.0 ± 1.3 mU mg^−1^) as compared to the wild type (16.5 ± 1.6 mU mg^−1^). Moreover, the absence of GlpR in *P. putida* Δ*upp* Δ*glpR*::Ptac*-gdhA* shortened the lag-phase during growth in glycerol minimal medium to a comparable extent as reported previously (Nikel et al., 2015; data not shown). We followed the method by Graf and Altenbuchner (2011) for markerless gene deletion and assumed comparable genetic stability for gene replacements. We have not tested long-term genetic stability, a requirement for establishing a fermentative large-scale process. Transformation of this strain with pEV1-*mgsABC-gmaS* yielded *P. putida* strain NMG3 (Figure 1). As consequence of *glpR* deletion and the second chromosomal *gdhA* copy transcribed from Ptac, production of 3.9 ± 0.1 g L^−1^ NMeGlu with a volumetric productivity of 0.08 g L^−1^ h^−1^ and a yield of 0.2 g g^−1^ glycerol by *P. putida* NMG3 resulted.

### Fed-batch mode production of NMeGlu from glycerol in bioreactors

Industrial applications require stable production at larger scale. A fed-batch cultivation with the NMG3 strain was performed to test the robustness of NMeGlu production by the metabolically engineered *P. putida* strain NMG3 under more controlled conditions.

**Figure 3:**
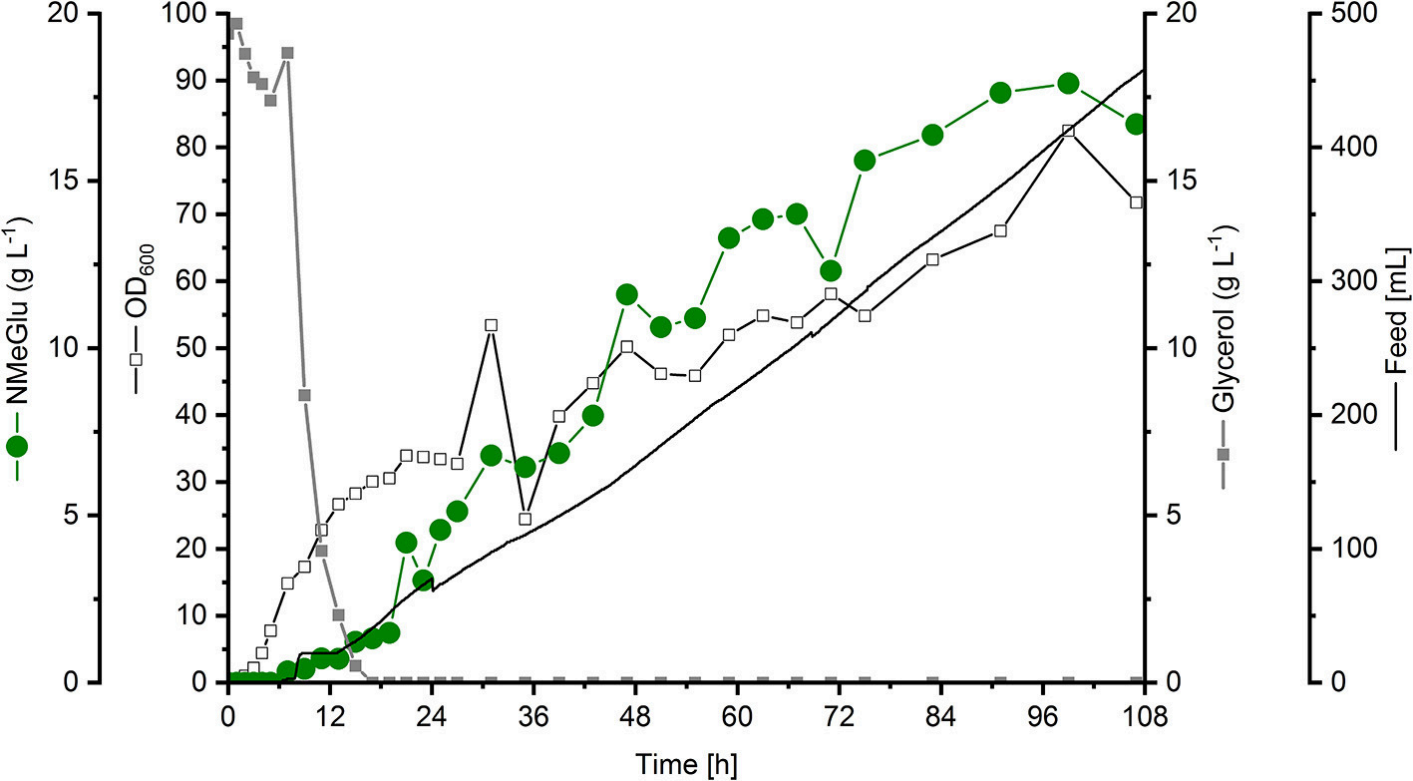
Fed-batch cultivation of *P. putida* NMG3 using HSG medium supplemented with 100 mM MMA. Starting with an initial volume of 2 L, the feed solution was coupled to the rDOS value. The feed volume is indicated by the black line. After 24 and 48 h 100 mm MMA was added. The biomass formation (black open squares) and concentrations of glycerol (gray closed squares) and NMeGlu (green closed circles) are shown. All concentrations, the feed volume and the biomass formation were related to the initial volume.

Starting with an initial volume of 2.0 L in the bioreactor, 460 mL feeding solution containing 600 g L^−1^ glycerol, 100 g L^−1^ yeast extract and 1 g L^−1^ MgSo${{}_{4}}^{*}$ H_2_O was added depending on the rDOS as explained in section Fed-batch cultivation *P. putida* KT2440 strains. At the end of the fed-batch phase [99 h (overall cultivation time 107 h)] in total 316 g glycerol were added with no residual substrate concentration in the cultivation broth. Only traces of the precursor l-glutamate accumulated (< 0.1 g L^−1^). The addition of MMA after 24 and 48 h of cultivation resulted in an interim reduction of biomass formation (see course of the OD_600_ in Figure 3). Probably, the cells had to adapt first to the modified cultivation conditions.

The newly constructed *P. putida* strain NMG3 produced NMeGlu to a final titer of 17.9 g L^−1^ with a volumetric productivity of 0.13 g L^−1^ h^−1^ and a yield of 0.11 g g^−1^ glycerol (Figure 3).

## Discussion

To the best of our knowledge, this is the first report of fermentative NMeGlu production. The recombinant *P. putida* KT2440 strain engineered here proved to be suitable for efficient NMeGlu production in fed-batch bioreactor cultivations.

**Figure 4:**
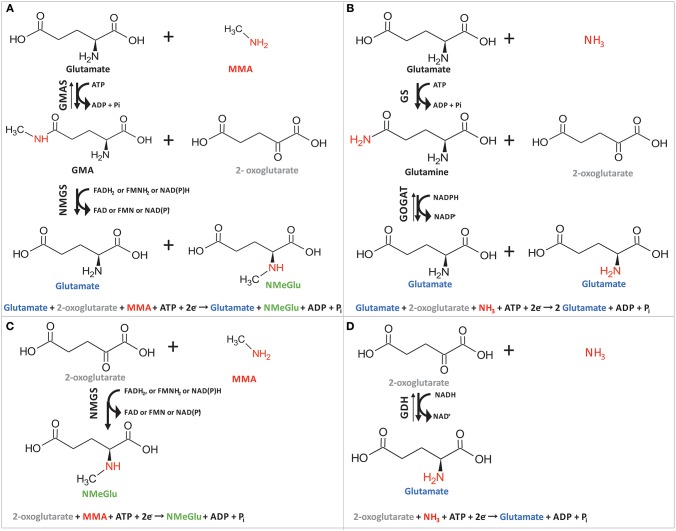
Schematic overview of the two-step cascade of GMAS/NMGS **(A)** in comparison to the GS/GOGAT mechanism **(B)** and the proposed one-step reaction of NMGS **(C)** in comparison to the GDH **(D)** reaction. The γ-glutamylmethylamide synthetase (GMAS) amidates glutamate at C^5^ position followed by a transfer of the *N*-methylgroup of l-glutamine to 2-oxoglutarate catalyzed by *N*-methylglutamate synthase (NMGS) **(A)**. Glutamine synthetase (GS) amidates l-glutamate to l-glutamate and the glutamate synthase catalyzes intermolecular transfer of the amino group to 2-oxoglutarate **(C)**. NMGS methylamidates 2-oxoglutarate at C^2^ position to NMeGlu **(C)**. Glutamate dehydrogenase (GDH) reductively aminates 2-oxoglutarate for l-glutamate **(D)**.

Recently, we have shown efficient fermentative production of another *N*-methylated amino acid, namely, *N*-methylalanine (Mindt et al., 2018). Although high titers (17.9 g L^−1^ NMeGlu as compared to 31.7 g L^−1^ NMeAla), yields (0.1 g NMeGlu per g glycerol as compared to 0.71 g NMeAla per g glucose), and volumetric productivities (0.13 g L^−1^ h^−1^ for NMeGlu as compared to 0.35 g L^−1^ h^−1^ for NMeAla) were reached for both methylated amino acids, the chosen pathways were different. In the case of NMeAla, the IRED DpkA was used for reductive methylamination of the oxo acid pyruvate, while in the pathway chosen here GMAS catalyzed ATP-dependent methylamidation of the γ-carboxyl group of l-glutamate to γ-glutamylmethylamide. In the subsequent reaction, NMGS is proposed to catalyze transfer of the *N*-methyl group from γ-glutamylmethylamide to 2-oxoglutarate yielding NMeGlu and l-glutamate (Gruffaz et al., 2014) (Figure 4A). Thus, this reaction cascade resembles the well understood glutamine synthetase (GS)/ glutamate synthase (GOGAT) cascade (Figure 4B) of several bacteria including *Pseudomonas fluorescens, E. coli* or *C. glutamicum* (Meers et al., 1970; Merrick and Edwards, 1995; Tesch et al., 1998). Moreover, sequence comparison of GMAS (EC 6.3.4.12) and NMGS (EC 2.1.1.21) via protein-protein BLAST (blastp) within bacterial taxa (taxid:2) revealed high similarity of GMAS to glutamine (Table 3). In the GS/GOGAT cascade, GS (EC 6.3.1.2) amidates l-glutamate to l-glutamine in an ATP-dependent manner using ammonia, which resembles ATP-dependent methylamidation of l-glutamate to γ-glutamylmethylamide by GMAS (Figure 4). GOGAT (EC 1.4.1.13) transfers the amide group of l-glutamine to 2-oxoglutarate yielding two molecules of l-glutamate in a similar manner as NMGS transfers the methylamide group of γ-glutamylmethylamide to 2-oxoglutarate yielding l-glutamate and NMeGlu (Figure 4). The GOGAT reaction requires NADPH as cofactor. Since the *N*-methylglutamate pathway of methylotrophic bacteria has not yet been elucidated in detail, the nature of the redox cofactor of NMGS remains to be identified (amongst other, NADH, NADPH or reduced ferrodoxin are possible candidates). The GS/GOGAT-like two-step pathway involving GMAS/NMGS yielded the highest NMeGlu titer, however, we also observed NMeGlu production in strains possessing NMGS, but lacking GMAS (0.2 ± 0.1 g L^−1^, Table 2). A plausible explanation for this 2.5 fold lower NMeGlu formation may be reductive methylamination of 2-oxoglutarate to NMeGlu as proposed by Gruffaz et al. (2014). A minitransposon screen and complementation studies revealed that GMAS is essential for growth of *M. extorquens* DM4 with MMA as sole source of carbon and energy, but not with MMA as sole nitrogen source (Gruffaz et al., 2014). According to the proposal, NMGS may catalyze reductive methylamination of 2-oxoglutarate (Figure 4C). In line with this notion, GOGAT of *B. subtilis* and *E. coli* has been described to catalyze reductive amination of 2–oxoglutarate to l-glutamate as side activity (Mäntsälä and Zalkin, 1976a,b; Matsuoka and Kimura, 1986). Further, this one-step reaction resembles the well understood glutamate dehydrogenase (GDH, EC 1.4.1.2) activity, where 2-oxoglutarate is converted to glutamate in an NADH dependent manner using free ammonium (Figure 4D). In principle, reductive (methyl)amination of 2-oxoglutarate by NMGS from *M. extorquens* and by GOGAT of *B. subtilis* resembles formation of NMeGlu from l-glutamate and MMA (Pollock and Hersh, 1971, 1973). Clearly, future in-depth biochemical analysis of GMAS and NMGS will have to unravel the mechanism of NMeGlu formation in *M. extorquens*.

**Table 3:** Protein-protein sequence comparison.

Used protein sequence^*^^#^	**Blastp-best hit**	**Identity (%)**	**Conserved protein domain family**
GMAS (*gmaS*; METDI2327)	type III glutamate-ammonia ligase *Bosea vaviloviae* (WP_069689945.1, [432 aa])	78	Family: glutamine synthetase, type III
NMGS I (*mgsA*; METDI2324)	glutamine amidotransferase *Rhizobiaceae bacterium* (WP_114956843.1, [300 aa])	72	Glutamine amidotransferases class-II (Gn-AT)_GlxB-type; Glutamate synthase domain 1 (GltB1)
NMGS II (*mgsB*; METDI2325)	protein glxC *Ancylobacter rudongensis* (WP_091437349.1, [225 aa])	80	Glutamate synthase domain 3 (GltB3)
NMGS III (*mgsC*; METDI2326)	FMN-binding glutamate synthase family protein *Novosphingobium acidiphilum* (WP_028640033.1, [442 aa])	88	Glutamate synthase (GltS) FMN-binding domain; Glutamate synthase domain 2 (GltB2)

*Protein sequences of GMAS, NMGS (I, II, III) of M. extorquens DM4 were analyzed within the bacterial taxid:2 using the algorithm blastp (Altschul et al., 1990). The best hits for each protein BLAST (lowest e-value) are shown. The predicted protein function, conserved protein domain family and the sequence identity are shown*.

* *Amino acid sequence analysis was performed with blastp (https://blast.ncbi.nlm.nih.gov/Blast.cgi)*.

# *Search within the bacterial taxid:2, excluding the genus Methylobacterium*.

The fermentative production of NMeGlu may benefit from improved supply of the precursor l-glutamate. In *P. putida*, l-glutamate dehydrogenase encoding gene *gdhA* is repressed by NtrC upon nitrogen limitation (Hervás et al., 2010), a condition unlikely to occur in the nitrogen rich media used here. Here, we have shown that expression from a second *gdhA* copy under control of the constitutive promoter Ptac and independent of NtrC increased NMeGlu production. However, the GDH activity only increased marginally when compared to the wild type, thus, further boosting of *gdhA* expression by choice of different promotors or ribosome binding sites may help to increase NMeGlu production even more. Another strategy to increase l-glutamate availability has been used in the well-studied l-glutamate producer *C. glutamicum* and occurred as consequence of reduced flux via the 2-oxoglutarate dehydrogenase complex, a TCA cycle reaction competing with l-glutamate dehydrogenase common substrate 2-oxoglutarate (Börmann et al., 1992; Kawahara et al., 1997; Asakura et al., 2007; Jorge et al., 2017). Since 2-oxoglutarate is precursor of l-glutamate and substrate of the NMGS reaction, this strategy has the potential to further increase NMeGlu production. The application of a synthetic promotor library could increase the plasmid-based gene expression as well as the expression of the integrated GDH. Elmore and co-workers developed a synthetic promotor library for rapid modifications for the application in *P. putida* KT2440. Based on the artificial promotor Ptac, which was used as well in this study, several UP elements, −35 sequences, and −10 sequences, as well as different ribosomal binding sites were tested and analyzed by using the reporter gene mNeonGreen (Elmore et al., 2017). This system could be further developed for the improvement of NMeGlu production in subsequent studies and promotor activities could be alternated for efficient plasmid-based and genome-based gene expression. Tests on genetic stability are required for establishing a fermentative large-scale process. In addition to further strain development, optimization of the fed-batch cultivation may lead to higher NMeGlu production. For example, further upscaling must include optimization of the process with respect to the feed composition to lower production costs (*i.a*. avoiding yeast extract) and increase space-time yields.

Alternative feedstocks are applied in industrial biotechnology processes to decrease the use of the dominant feedstock glucose, which has competing uses in feed and food industries. Several biotechnological production hosts including *P. putida, E. coli, C. glutamicum*, and yeast are metabolically engineered for access to alternative carbon and energy sources (Wendisch et al., 2016). Although the focus mostly lay on pentose sugars present in lignocellulosic hydrolysates, access to glycerol, which is readily available from biodiesel production, plays an important role. The natural ability of *P. putida* to use glycerol as carbon and energy source is an inherent advantage also used in this study. Interestingly, *P. putida* grows to higher biomass concentrations with glycerol than with glucose (Escapa et al., 2013). The application of *P. putida* as glycerol utilizing host benefits from its tolerance to impurities present in crude glycerol, which may pose challenges for production with other hosts (Meiswinkel et al., 2013; Nguyen et al., 2013). Therefore, *P. putida* has been used for glycerol-based production of oxo-carboxylates (Wang et al., 2015), polyhydroxyalkanoates (Wang and Nomura, 2010; Poblete-Castro et al., 2014; Wang et al., 2014) or *p*-hydroxybenzoate (Verhoef et al., 2007). The molecular reason why glycerol is preferred over glucose is still not solved. Both substrates have identical degrees of reduction, but glucose is partially oxidized to gluconate and ketogluconate. Moreover, a transciptomic study revealed that genes involved in the general stress response show higher RNA levels during growth with glucose as compared to growth with glycerol (Nikel et al., 2014a).

Taken together, expression of genes from *M. extorquens* enabled NMeGlu production by *P. putida* GRAS strain KT2440 via GMAS and NMGS. Metabolic engineering of the precursor supply and derepression of glycerol catabolism led to efficient production of NMeGlu to a final titer of 17.9 g L^−1^ with a volumetric productivity of 0.13 g L^−1^ h^−1^ and a yield of 0.11 g g^−1^ glycerol.

## Author contributions

VW conceived the study. All the authors planned the experiments. MM and TW performed the experiments. MM, TW, and JR analyzed experimental data. MM and TW, drafted the manuscript. VW finalized the manuscript. All the authors agreed to the final version of the manuscript.

### Conflict of interest statement

The authors declare that the research was conducted in the absence of any commercial or financial relationships that could be construed as a potential conflict of interest.
