# Supplementary material for: Fermentative Production of N-Methylglutamate From Glycerol by Recombinant Pseudomonas putida
Source: Front Bioeng Biotechnol. 2018 Nov 9;6:159. doi: 10.3389/fbioe.2018.00159 (PMC6237917; doi:10.3389/fbioe.2018.00159)
Supplement: Supplementary file 1 [file Data_Sheet_1.pdf]

SUPPLEMENTARY DATA

Fermentative production of *N*-methylglutamate from glycerol by recombinant *Pseudomonas putida*

Melanie Mindt[a,#], Tatjana Walter[a,#], Joe Max Risse[b] and Volker F. Wendisch[a,*]

[a] Genetics of Prokaryotes, Faculty of Biology & CeBiTec, Bielefeld University, Bielefeld, Germany

[b] Fermentation Technology, Technical Faculty & CeBiTec, Bielefeld University, Bielefeld, Germany

[#] both authors contributed equally.

*Corresponding author: Volker F. Wendisch, Chair of Genetics of Prokaryotes, Faculty of Biology & CeBiTec, Bielefeld University, Germany; phone: +49-521-106 5611; fax: +49-521-106 5626; volker.wendisch@uni-bielefeld.de

Supplementary Table S1: Oligonucleotides used in this work.

Supplementary Table S2: Protein-protein sequence comparison within *Pseudomonas putida* KT2440.

Supplementary Figure S1: Protein gel analysis of different recombinant *P. putida* KT2440 strains.

Supplementary Table S1: Oligonucleotides used in this work.

Name	Sequence 5’-3’	Species
**P1**	CCTGCAGGGGCCGGCCGTTTAAACCTAGGAGGATTCGTCATGTGCGGTATTGTCGGACT	*M. extorquens* DM4
**P2**	CCCTACTCTCGCGTGCTCGAGTCAGCAGTCGAGCGTGGTGTC	*M. extorquens* DM4
**P3**	CCCTACTCTCGCGTGCTCGAGTCAGATGCCGGTCTTGCC	*M. extorquens* DM4
**P4**	GATTACGCCAAGCTTGCATGCCCACAAGCCCTCTACACGTTC	*P. putida* KT2440
**P5**	GCTGGCTCAGGGCGTGGTCTGAGGGCTGGTGGGTGCATGCCG	*P. putida* KT2440
**P6**	TTGACAATTAATCATCGGCTCGTATAATGCTAGGAGGATTCGTCATGTCTACCATGATCGAATC	*P. putida* KT2440
**P7**	CGGCATGCACCCACCAGCCCTCAGACCACGCCCTGAGCCAG	*P. putida* KT2440
**P8**	CATTATACGAGCCGATGATTAATTGTCAAGGGCGGTCCTTTGGGGCTGC	*P. putida* KT2440
**P9**	GCACGATGGCCGCTTTGGTCCCGTTCCTGTTGATGAACACG	*P. putida* KT2440
**P10**	GTGCCGTGGCCAACAACTTC	*P. putida* KT2440
**P11**	GCCAGGTCGTCTTTTTCGCAAAGG	*P. putida* KT2440

Supplementary Table S2: Protein-protein sequence comparison within *Pseudomonas putida* KT2440. Protein sequences of GMAS, NMGS (I, II, III) of *M. extorquens* DM4 were analyzed within *Pseudomonas putida* KT2440 (taxid:160488) using the algorithm blastp (Altschul et al., 1990). The best hits for each protein BLAST (lowest e-value) are shown. Genes, with predicted function in KT2440, were analyzed on the amino acid sequence. Pairwise sequence alignments of GMAS and NMGS against predicted proteins using the algorithm EMBOSS NEEDLE (Chojnacki et al., 2017) were performed.

**Used protein sequence* #**	**Blastp - Best hit**	**BLAST Identity**	**Identified genes in *P. putida* KT2440**	**Amino acid sequence alignment (EMBOSS Needle) – Identity~**
GMAS (*gmaS*; METDI2327)	MULTISPECIES: glutamine synthetase [*Pseudomonas*]	29%	PP_5046<br>PP_3148<br>PP_4547<br>PP_4399	28.2 %<br>28.2 %<br>28.7 %<br>26.5 %
NMGS I (*mgsA*; METDI2324)	MULTISPECIES: class II glutamine amidotransferase [*Pseudomonas*]	33%	PP_2179<br>PP_5298	12.7 %<br>15.1 %
NMGS II (*mgsB*; METDI2325)	No hits	-	-	-
NMGS III (*mgsC*; METDI2326)	MULTISPECIES: FMN-binding glutamate synthase family protein [*Pseudomonas*]	31%	PP_5075<br>PP_5076<br>PP_0269<br>PP_1060	9.1 %<br>8.2 %<br>22.4 %<br>23.2 %

* Amino acid sequence analysis was performed with blastp (https://blast.ncbi.nlm.nih.gov/Blast.cgi)
# Search within *Pseudomonas putida* KT2440 (taxid:160488)
~ Pairwise sequence alignment using EMBOSS NEEDLE (https://www.ebi.ac.uk/Tools/psa/emboss_needle/)

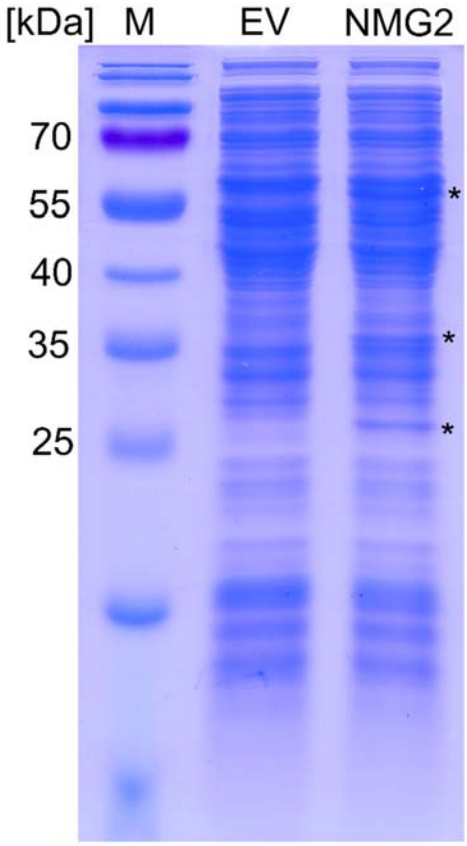

Supplementary Figure S1: Protein gel analysis of different recombinant *P. putida* KT2440 strains. Cultures were grown in M12 minimal medium supplemented with 10 g L^{-1} glucose for 24 h. Expected protein masses: NMGS subunit A: 33 kDa, NMGS subunit B: 24 kDa, NMGS subunit C: 47 kDa and GMAS: 48 kDa. Additional protein bands are within the expected size range and marked with an asterix. M: Page ruler prestained protein ladder 10-180 kDa (Thermo Fischer scientific, USA). EV: *P. putida*(pEV1). NMG2: *P. putida*(pEV1-*mgsABC-gmaS*).
